# Supplementary material for: Are carbon emissions trading and green financial instruments synergistic? -Comprehensive quantitative research based on content analysis
Source: PLoS One. 2024 Mar 7;19(3):e0298601. doi: 10.1371/journal.pone.0298601 (PMC10919601; doi:10.1371/journal.pone.0298601)
Supplement: S1 Data — (ZIP) [file pone.0298601.s001.zip › Data/地级市Z_F工作报告.zip!/║╙─╧/╞╜╢Ñ╔╜╩╨/└┤╫╘╣1⁄2╓┌║┼í╛┬φ┐╦╩2╛▌═°í┐_2015.docx]

各位代表！

2014年，我市各项工作在异常困难形势下能取得新的进展，这是省委、省政府和市委正确领导的结果，是市人大、市政协积极支持的结果，是全市上下团结拼搏的结果。回顾过去一年的工作，我们最感到欣慰的成绩是，初步扭转了近几年经济增速连续下滑的被动局面，经济运行出现回升。最令人振奋的事情是，郑万高铁经过矢志不渝的争取，最终由东线方案调整为西线方案，并在城乡一体化示范区（新区）和郏县设站，必将极大提升我市交通区位优势。最让人难忘的场面是，面对旱魔，全市广大干部群众同心协力、众志成城，打赢了抗旱减灾这场硬仗。最感受深刻的体会是，改革、开放、创新是迎接挑战、攻坚克难的最大法宝，只要敢啃硬骨头、敢于涉险滩，就能不断把改革开放创新的红利转化为发展新动能和民生新福祉。最值得弘扬的精神是，困难面前勇于担当，咬定青山不放松，锲而不舍抓落实，使一批事关全局和长远的大事难事最终办成或取得突破性进展。

成绩来之不易，经验弥足珍贵。在此，我代表市人民政府，向工作在全市各条战线的广大工人、农民、知识分子、干部、驻平解放军指战员、武警官兵和公安民警致以崇高的敬意！向关心、支持我市现代化建设的各界人士和国际友人、港澳台同胞、海外侨胞表示衷心的感谢！

在肯定成绩的同时，我们也清醒地看到，我市经济社会发展中仍存在一些不容忽视的矛盾和问题。一是经济增速位次靠后。生产总值增速虽有回升，但仍处于全省后列。二是结构性矛盾依然突出。现代服务业规模小，能源原材料工业占比高，战略性新兴产业和高技术产业薄弱，非公有制经济发展相对滞后。三是经济发展后劲不足。项目储备不够，投资总量依然偏低。四是各种风险隐患增多。民生改善任务仍很繁重，社会矛盾多样多发，近期又出现了因非法集资所引发的不稳定因素。五是政府自身建设仍需加强。个别部门职能转变不到位，一些干部思想观念落后、改革开放意识不强、工作作风不实，缺乏面对落后的危机感、奋起直追的紧迫感、造福一方的责任感。对此，我们一定高度重视、认真解决，决不辜负全市人民的期望和重托！

二、2015年工作总体要求和主要目标

今年是全面完成“十二五”规划的收官之年。当前我国经济发展进入新常态，呈现速度变化、结构优化、动力转换的特点，我市作为资源型城市，受到的冲击更严重，稳增长调结构难度更大，但仍处于可以大有作为的重要战略机遇期，经济平稳回升的趋势没有改变，资源比较优势依然明显，传统产业改造提升空间巨大，装备制造、生物医药、电子信息等战略性新兴产业逐步崛起，现代服务业发展加快，新的经济增长点正在形成。全国老工业基地调整改造规划和资源型城市可持续发展规划的深入实施，为我市争取项目、调整转型将提供更多支持。郑万高铁、沙河复航、城市轻轨、孟平铁路复线、大西环等重大基础设施的规划建设，也必将极大提升我市综合竞争优势。只要我们善于把握机遇、积极应对挑战，就一定能在困难中争取主动、放大优势、赢得未来，让平顶山经济在新常态下迈上新台阶、实现新突破。

政府工作的总体要求是:全面贯彻党的十八大和十八届三中、四中全会精神，深入学习贯彻习近平总书记系列重要讲话精神，按照中央、省委、市委经济工作会和市委八届八次全会部署，主动适应经济发展新常态，坚持转中求进工作总基调，以提高发展质量和效益为中心，着力调整经济结构，着力深化改革开放，着力强化创新驱动，着力夯实基础支撑，着力化解各类风险，着力加强民生保障，持续推进转型提速、发展提质、环境提优、幸福提升，努力在全面建成小康社会征程上迈出更加坚实的步伐。

经济社会发展的主要预期目标是:在优化结构、提高质量、改善环境的基础上，生产总值增长8%左右；公共财政预算收入增长9%；全社会固定资产投资增长17%；社会消费品零售总额增长13%；进出口总值增长9%；实际利用外商直接投资增长8%；城乡居民收入增长与经济增长同步；居民消费价格涨幅控制在3%左右；节能减排和环境质量改善完成省下达任务。

实现上述要求和目标，我们必须主动适应经济发展新常态，清醒认识增速换挡的必然性、结构调整的艰巨性、动力转换的长期性，坚决破除资源过度依赖思想，坚决破除单纯依赖传统产业观念，坚决破除经济周期性变化依赖思维，坚决破除路径依赖心态，更加注重经济结构优化升级，更加注重加大改革创新力度，更加注重保障和改善民生，更加注重改进工作方式方法，按照经济规律、自然规律和社会规律来谋划发展、推动工作，真抓实干、奋力拼搏，努力开创各项工作新局面！

三、2015年重点工作

今年重点抓好九个方面的工作：

（一）持续扩大有效需求，促进经济平稳健康发展。充分发挥投资的关键作用、消费的基础作用和服务的保障作用，挖掘需求潜力，拓展需求空间，促进经济平稳增长。

科学推进新型城镇化。认真落实科学推进新型城镇化的实施意见，加快新型城镇化进程，城镇化率提高1.5个百分点。推进农业转移人口市民化。落实户籍制度改革政策，把市区和县城作为承接农村人口转移的主体，优先解决一批已进城就业定居的农民工落户，成建制解决一批城中村居民转户，推动一批农村富余劳动力有序转移，对未落户的农业转移人口建立以居住证为载体的基本公共服务提供机制，切实保障进城农民原有权益，消除农民进城的后顾之忧。优化城市发展空间布局。健全空间规划体系，围绕“一点一线”，完成高铁站及周边规划设计，开展城市轻轨沿线规划设计，着力打造城市发展新的增长极。完成快速路网、地下空间开发利用等规划编制，推进市县“多规合一”。提高城镇综合承载能力。全面推进城乡一体化示范区建设，抓好平安大道西延、平郏快速通道、平宝路改线、焦庄水厂等57个基础设施项目建设，推进大西环和平叶快速通道前期工作，开工建设1所高中、1所初中和2所小学，加快华侨医院等项目进度。积极推进城乡一体化先行先试，努力在公共设施、公共服务等方面实现均等化。改造提升老城区，抓好南环路东西延、东环路南延、集中供热管网扩建一期等47个基础设施项目，完成鹰城广场提档升级、白鹭洲国家城市湿地公园改造，开工火车站区域综合改造项目，加快湛南老工业区整体搬迁改造。统筹规划建设城市公交、医院、学校等公共服务设施，新增260台新能源公交车。实施城市标准化管理，巩固创卫成果，确保获得国家卫生城市命名。启动国家智慧城市试点申报工作。健全“双违”整治长效机制。加强县城和中心镇建设，增强吸纳承载能力。支持独立工矿区改造搬迁试点建设。促进房地产市场健康发展。创新城建体制机制。以维护被征地群众合法权益为根本，依法推进市政重点工程征地拆迁安置工作，并作为考核县(市、区)干部和乡(镇、办)主要领导的重要内容。以国开行、公积金贷款支持棚改项目为抓手，推进城建融资和土地收储，完成土地收储5500亩。积极融入省新型城镇化发展基金，探索设立我市子基金。

努力扩大投资需求。突出抓好320个市级重点项目建设，带动全社会固定资产投资完成1450亿元。其中，农业项目26个，年度投资32.7亿元。重点抓好康龙现代农业示范园区、舞钢瑞祥粪污处理沼气提纯工程等项目。工业项目144个，年度投资271亿元。重点抓好平煤神马集团己二酸己内酰胺一期、力帆树民摩托车及电动车工业园、中材环保高端环保装备产业化等项目。服务业项目58个，年度投资104.5亿元。重点抓好应山主题公园、市现代物流产业园一期、宝钢装配式绿色建筑产业园等项目。交通项目11个，年度投资38.5亿元。重点抓好郑万高铁平顶山段、孟平铁路复线电气化改造、沙河复航平顶山段等项目。城建项目69个，年度投资144.4亿元。重点抓好湛河综合治理、东王营安置小区等项目。社会事业项目12个，年度投资9亿元。重点抓好矿山医疗救助中心综合楼、市四十一中异地重建等项目。同时，抢抓国家在铁路、水利、农村公路、保障性安居工程等领域扩大投资的机遇，抓紧谋划上报一批重大项目，争取列入国家、省计划盘子。落实鼓励和促进民间投资的政策措施，着力在基础设施和社会事业等领域引进社会资本参与建设和经营。

积极扩大消费需求。进一步提高中低收入居民收入，调整机关事业单位人员工资，多渠道增加农民收入，提升消费能力。加强与国家养老健康、信息、旅游、住房、节能环保、教育文化等6大领域消费工程衔接，抓好农村超市、城市便民连锁网店、电子商务公共服务平台建设，加快培育新的消费增长点。大力优化消费环境，整顿和规范市场秩序，依法维护消费者权益。

持续深化企业服务。健全企业服务长效机制，重点解决制约企业发展的“卡脖子”问题。深入推进银企合作、中小企业金融超市、金融服务进产业集聚区等活动，开展中小企业房产、地产确权颁证专项行动，规范完善担保服务体系，着力解决中小企业融资难问题。规范行业协会涉企收费，有偿中介服务引入竞争机制。实施建设用地指标差异化、精细化管理，保障重点项目用地需求。

（二）持续调整产业结构，加快产业转型升级步伐。把握产业发展规律，推动工业、服务业协调发展，促进产业融合发展，向结构调整要增长、要质量、要效益。

推动工业转型发展。进一步扩大优质增量、调整优化存量，加快构建以战略性新兴产业为引领、传统优势产业活力凸显的现代工业发展新格局。改造提升传统产业。综合运用退城进园、承接转移、延伸链条等手段，加快对能源、化工、冶金、建材等传统优势产业进行“脱胎换骨式”改造，重点抓好平煤神马集团22万吨烧碱异地搬迁、天宏公司120万吨焦化异地搬迁、平煤神马集团100兆瓦光伏发电等项目建设，加快建设国家重要的新型能源化工基地。发挥舞钢公司产业和技术优势，加快技术创新和装备升级。大力发展轻工食品、纺织服装等产业，主动和行业龙头企业对接合作，走深加工、高端化路子，提高产品质量和档次。培育壮大战略性新兴产业和高成长性制造业。依托平高集团、隆鑫机车、平煤机等企业，培育壮大装备制造产业，重点抓好平新机电装备产业园、隆鑫机车产业园、正合汽配产业园、南车集团高端矿用设备等项目建设，加快建设国家重要的现代装备研发制造基地。依托圣光集团、河南真实生物科技、佳瑞高科等企业，培育壮大生物医药产业，重点抓好国际医疗器械孵化园、FNC抗艾滋病毒新药研发推广、国内培南类抗生素研发生产基地等项目建设。依托中兴科技、尧谷光电、东陆高科等企业，培育壮大电子信息产业，重点抓好中兴通讯手机及配件产业园、尧谷光电触控显示模组等项目建设。依托宝丰翔隆等企业，培育壮大不锈钢产业，重点抓好翔隆不锈钢150万吨板材等项目建设。加快产业集聚集群发展。以晋星升级为目标，编制完善产业集聚区产业发展规划，实现管理体制、规模水平、招商引资、综合配套功能的完善和提升。年度完成投资650亿元，增长17%以上；主营业务收入突破1000亿元，增长10%以上；6家产业集聚区达到一星标准、3家达到门槛标准，争创国家、省经济技术开发区或高新技术开发区。把培育壮大五大产业集群摆在更加突出位置，加快编制化工等专业园区产业发展规划，推动同类和关联项目集中布局，逐步在各县(市、区)形成一批拥有2家以上行业龙头企业、20家以上关联配套企业，主营业务收入过百亿的特色产业集群。

加快发展服务业。以实施服务业“62231”发展计划为抓手，持续抓好文化旅游、现代物流、金融保险、现代商贸、信息服务、养老和健康服务等六大主导产业，确保服务业增加值增长8%以上。突出龙头带动。重点支持20家骨干企业，加快培育一批具有较强市场影响力的知名服务品牌。突出发展旅游业，编制城市旅游等3个规划，加快大香山风景名胜区、舞钢龙凤湖度假区等重点景区建设，力争2家景区晋升4A级景区、3家晋升3A级景区，努力建设海内外知名的旅游目的地。强化项目支撑。滚动扶持30个市场潜力大、经济效益好、带动能力强的项目，推进G311国道改线、尧山-大佛景区大型停车场等项目建设，建成义乌国际商贸城等项目，加快红星美凯龙、苏宁广场等项目前期工作。狠抓载体建设。大力推进商务中心区和特色商业区建设，健全“两区”工作机构和推进机制，完成投资70亿元。争取2个特色商业区进入全省前40名，市商务中心区进入全省前30名。扶持培育10个有一定基础的特色园区。推动中兴会展园尽快落地。规划建设区域性养老健康产业发展示范园区，鼓励圣光集团发展健康养老产业。加大政策支持。落实支持服务业发展各项措施，全面清理限制性规定，降低医疗卫生、教育培训等领域准入门槛，扩大购买社会服务规模。

促进产业融合发展。顺应制造业发展从硬件向软件、解决方案等无形服务转变的趋势，鼓励引导制造业服务化，推动其向研发、设计、物流、营销、品牌推广和系统集成等方面延伸。用信息技术改造提升传统产业，促进云计算、大数据、物联网等与现代制造业结合，加快生产设备数字化改造，推动信息技术在现代物流、工业设计等服务业领域广泛应用。

（三）持续发展现代农业，巩固“三农”基础地位。深入推进种植规模化、养殖标准化、经营产业化、生产机械化和美丽乡村建设，促进农业发展方式转变。大力发展现代农业。持续实施高标准粮田“百千万”建设工程和粮食高产创建，新建高标准粮田13万亩。积极发展家庭农场、农民合作社、产业化龙头企业等新型农业经营主体，新增市级以上龙头企业20家、农民合作社300 家、家庭农场100家，培育1~2个示范性产业化集群。研究出台以畜牧业为龙头、农牧结合的现代生态循环农业试验区建设实施意见，支持建立循环农业研究院和试验室，整合各项农业投入向循环农业倾斜。发展乡村休闲旅游和城市近郊都市生态农业，努力打造2~3个都市生态农业示范园区。推进“鹰城农业”品牌建设。加快投融资平台、农产品追溯平台和电子商务平台建设。加强农业基础能力建设。抓好应急水源、病险水库除险加固、北汝河河道治理、昭平台灌区续建、南水北调配套等重点水利工程建设。加快农业科技创新和重大技术推广，支持市农科院省韭菜工程技术研究中心、博士后研发基地建设。抓好农网改造升级工程和农村低电压治理。加强气象现代化建设，建好气象科技园。提升农业机械化水平，新增农机总动力5万千瓦。不断深化农村改革。规范引导农村土地经营权有序流转，加快农村土地承包经营权确权登记颁证，完成农村宅基地使用权和集体土地建设用地使用权确权登记发证工作，探索建立农村宅基地有偿退出机制。支持畜牧合作总社发展壮大。创新供销社组织体系和服务机制。鼓励设立财政参股、控股的混合所有制涉农担保公司。建立农村产权交易市场，探索建立农村土地信托中心。加强对小农水项目投入绩效评估，完成国家级小型水利工程管护改革试点。分类推进新农村建设。完善提升新农村建设规划，重点支持规模较大、基础设施完善的67个中心村和与66个人地挂钩试点不重叠的村加快建设，力争在引导入住、拆旧复耕上取得突破。加强农村基础设施建设，新改建干线公路131公里、农村公路490公里，改造桥梁5310延米，再解决28.4万农村居民和5万农村师生饮水安全问题。深入开展改善农村人居环境活动，建设美丽乡村。

（四）持续完善创新体系，增强创新驱动发展能力。紧紧抓住科技与经济紧密结合这个核心，加快推进以科技创新为重点的全面创新。加强科技创新。集中市级财政科研经费，设立1000万元科技创新重大专项资金，支持企业发展高新技术。强化企业创新主体地位，创建省级工程技术中心3家、市级研发中心20家、产业创新联盟5个。推进企业与国内外同行、大型央企、科研院所、高等院校深度合作，引进或共建一批创新平台。鼓励企业通过海外并购、设立研发中心、购买专利技术增强创新能力，支持平煤神马集团组建炼焦煤资源利用国家工程技术研究中心，推动平煤神马集团进入国家自主创新领军企业试点。实施百项成果转化工程，优选能够显著促进我市转型发展的重大科技成果100项，推动创新成果向现实生产力转化。实施科技培育孵化工程，支持尼龙化工、高压电气等优势产业技术创新，培育国家级高新技术企业5家。积极创建国家知识产权示范市和科技进步先进市。加强商业模式创新。研究制定支持商业模式创新的政策措施，鼓励企业探索形式多样、市场接受的新型商业模式。把发展电子商务作为事关全局和长远的战略重点，推动与国内知名电商企业战略合作，加强电子商务平台、云计算中心建设，支持企业搭建电子商务集聚平台，开展电子商务示范企业创建活动。大力发展平台经济，推进大宗商品交易，促进有形市场与无形市场融合发展，线上交易与线下交易同步推进。加强创新创业人才培养引进。深入实施全民技能振兴工程和技能人才回归工程，完成各类职业技能培训14万人次。积极引进国内外创新人才，健全培养选拔、流动配置、激励保障等机制，培育市级科技创新杰出人才和创新型科技团队。

（五）持续建设文化强市，推动文化大发展大繁荣。挖掘文化资源优势，加快文化改革发展，进一步提升文化软实力。大力繁荣文化事业。深入开展省级公共文化服务体系示范区创建，实施舞台艺术送农民、广播电视户户通、“三馆一站”免费开放等文化惠民工程，组织开展好各类群众性文化活动。加强文化遗产保护，推进汝官窑遗址博物馆展示厅建设和应国墓地保护利用工作。做好省级全民健身示范城市创建工作，积极承办各类赛事，筹办好市九运会暨第三届全民健身大会。加快发展文化产业。认真落实重点文化产业园区和企业发展实施意见，推进文化与旅游融合发展，加快山顶公园、矿山遗址公园、宝丰文化创意产业园、郏县三苏文化产业园等项目建设，培育壮大工艺美术、魔术演艺、文化旅游等特色产业，着力打造一部魔术舞台情景剧。深化文化体制改革。深入推进国有经营性文化单位转企改制，完善电台、电视台合并和县（市、区）文化、广电、新闻出版三局合一后的管理机制。开展基层综合性文化服务中心建设试点。完成市广电传媒集团平台搭建工作。加强精神文明建设。培育和践行社会主义核心价值观，强化道德建设、诚信建设、法制建设和服务型机关建设。以创建全国文明城市为目标，广泛开展各类创建活动，完善志愿者服务体制机制，加强未成年人思想道德建设，提高城市文明程度。

（六）持续建设生态文明，着力打造美丽鹰城。大力推进节能减排和环境保护，努力使鹰城的天更蓝、地更绿、水更净。加大污染防治力度。继续实施蓝天工程，加快火电、钢铁、水泥行业脱硫脱硝改造、燃煤锅炉和机动车尾气治理，抓好城市建筑工地、城乡道路扬尘防治，做好农村面源污染治理和秸秆禁烧工作。全面推动水污染防治，加强白龟湖上游入湖河流绿化治理，推进新污水处理厂建设及老厂升级改造，完善城市污水收集系统。加大环保监察力度，严厉打击环境违法行为。强化能源资源节约集约利用。深入开展工业能效对标达标活动，加强大型公共建筑节能监管平台建设，抓好节约型公共机构示范单位创建，推进能源管理体系和重点用能单位在线监测，争取建设省级城市能源资源计量中心。加快实施节能环保技术装备产业化示范工程，做好工业锅炉窑炉、余热余压利用等节能技术改造，大力发展风电等清洁能源，积极开展清洁生产、再生资源回收和综合利用，提高能源资源利用效率。加强生态系统建设和修复。坚持最严格的耕地保护制度和水资源管理制度，加快白龟湖国家湿地公园规划建设，推进煤矿沉陷区治理和工矿废弃地整治。继续实施林业生态建设提升工程，抓好生态廊道网络、“百千万”防护林、城镇社区绿化美化等八大林业工程，完成造林5.3万亩。构建生态文明建设长效机制。完善能源消费和重点污染物排放总量预算管理制度，健全生态环境补偿、环境损害鉴定评估和责任追究等机制，实施节能量、排污权、碳排放权、水权交易制度。

（七）持续深化改革攻坚，健全科学发展体制机制。坚持“一跟进、两聚焦”，立足实际大胆实践，着力在重要领域和关键环节取得新突破。深化行政体制改革。全面完成市县政府机构改革和交通运输执法体制改革。理顺城乡一体化示范区、化工产业集聚区管理体制机制。继续取消、下放市级行政审批事项，全部取消非行政许可审批，减少前置审批，积极推行模拟审批、网上审批等新模式。加快建立政府权力清单、责任清单，探索实行负面清单管理，开展产业集聚区投资准入负面清单管理模式试点。创新行政审批后续监管方式，强化事中事后监管。深化市场主体改革。加快推进平燃公司股权转让、自来水公司和中房平顶山公司股份制改革，完成污水处理厂经营模式改革，完成星峰集团破产扫尾工作。启动国有资本投资公司和运营公司试点、职业经理人市场化选聘试点。大力发展混合所有制经济，支持平煤神马集团等国有企业控股参股民营企业改革，同时也鼓励非公有制企业参与国企改革，允许非国有资本参股国有资本投资项目。鼓励发电公司与本地企业开展直供电交易。深化财税金融改革。全面贯彻落实新预算法。稳步推进扩大“营改增”试点、煤炭资源税从价计征等税制改革。针对生产经营和纳税额度不匹配的行业企业，加大税务稽查力度，堵塞税收征管漏洞。启动涉企资金基金化改革试点，推进财政资金由无偿投入向股权投资转变。完善高新区、化工产业集聚区财税征管体制。引导中小企业在资本市场直接融资，力争10家以上企业在新三板或上海、天津股权交易机构挂牌上市。积极参与基础设施、战略性新兴产业、现代服务业等省产业投资基金和创业投资基金，鼓励保险资金参与我市发起设立的各类基金，支持市级投融资公司探索多元化融资模式。深化农村信用社改革，力争1~2家县级农信联社达到筹建农商行标准，实现舞钢、叶县村镇银行开业。加强金融生态环境考核评价工作，积极防范和化解金融风险。深化医药卫生体制改革。全面推进县级公立医院综合改革，深化基层医疗卫生机构改革，加快基本公共卫生服务均等化。开展城市区域和县域医疗联合体试点工作，提高医疗资源利用率。健全重特大疾病保障制度，全面实施城镇居民大病保险。深入推进社会办医。深化土地管理制度改革。扎实推进工矿废弃地复垦利用试点和人地挂钩试点工作。深化国有建设用地有偿使用改革，优化土地审批流程，着力解决批而未征、征而未供、供而未用等问题。同时，完成党政机关公务用车制度改革，深化社会事业、住房公积金等领域改革，稳步推进盐业体制改革。

（八）持续强化开放招商，提升开放型经济发展水平。发挥开放招商“一举求多效”的综合作用，推动全方位宽领域多层次开放。拓展开放领域。抓住国家放宽外商投资市场准入机遇，大力推动现代服务业、基础设施、节能减排、现代农业等领域开放招商，在现代物流、文化旅游、医疗、教育等方面引进一批重大项目。坚持以台湾和香港为重点，积极开展境外融资，吸引高端制造业转移。创新招商方式。围绕5大产业集群、9个省级产业集聚区和15个专业园区，充分发挥商会协会作用，大力开展精准招商、以商招商、产业链招商，力争在化工、电气装备、不锈钢制品、摩托机车、医疗器械、电子信息等产业取得突破性进展。继续开展招商引资专项督察，提高招商实效。促进外贸出口。在提升电气装备、特种钢材、尼龙化工、纺织品等出口优势的同时，扩大高新技术、农副产品等出口份额，支持企业开展境外商标注册、国际通行产品和体系认证。加强对外交流合作。鼓励煤炭、钢铁、水泥等企业走出去，扩大生存空间，化解过剩产能。发展对外承包工程与劳务合作，支持优势企业开展工程总承包。推进中德生态和环保合作试点项目，争取与德国劳恩堡市签署友好城市关系协议。优化开放软环境。建立健全客商投资项目审批代理制，进一步提高投资贸易便利化水平，打造亲商、安商、富商的良好环境，尽快完成由靠优惠政策招商向靠优质服务招商的战略性转变。

（九）持续保障改善民生，努力构建和谐社会。继续实施十项重点民生工程，让人民群众过上更好的生活。加强就业创业工作。重点抓好大学毕业生、农村转移劳动力、城镇困难人员、退役军人等群体就业，妥善解决结构性失业和化解产能过剩中下岗分流职工再就业等问题，城镇新增就业6万人以上，农村劳动力转移就业5万人。支持建设一批创业孵化示范园区，以创业促进就业。完善社会保障体系。继续提高企业退休职工和城乡居民基础养老金、优抚对象抚恤金和生活补助、城乡低保补助水平，推进机关事业单位养老保险制度改革。扩大社会保障覆盖范围，试行“五保合一”经办模式。适当提高农村五保供养标准，五保对象集中供养率提高到50%以上。抓好农村敬老院建设。搞好医疗救助与新农合、城镇居民医保的制度衔接，完善医疗救助“一站式”即时结算服务。全面实施临时救助制度，加强城乡“爱心救助超市”建设。加快市儿童福利院、未成年人保护中心和夕阳红老年公寓等项目建设。扎实推进保障性安居工程建设，新开工保障性住房1.45万套，基本建成1.11万套。加快老区建设，开展精准扶贫，再实现5万贫困人口稳定脱贫。全面发展社会事业。深入实施第二个学前教育三年行动计划；推动义务教育均衡发展，新建改扩建中小学23所；继续实施职教攻坚二期工程，围绕化工、装备制造等产业，开设相关专业，加强校企合作，提升办学水平；支持高等院校转型发展。完善公共医疗卫生服务体系，加快市第一人民医院新院区、第二人民医院新病房综合楼、中医院新院区、传染病医院新病房楼等项目建设，启动全市基层卫生云和市远程医学中心建设。积极开展全国幸福家庭创建试点工作，严格落实人口控制计划。持续发展慈善、残疾人、妇女儿童、红十字、老龄等事业。加强和创新社会治理。切实做好打击和处置非法集资及投资担保公司清理规范工作，坚决守住不发生区域性风险的底线。积极排查化解社会矛盾，加强行政复议、行政调解和仲裁工作，落实依法逐级走访、诉访分离和积案化解制度。加强公共安全管理和教育，组织开展各类突发事件应急处置演练。强化食品药品安全和安全生产监管，完善企业安全生产风险预控管理体系，推进安全生产打非治违专项行动，构建隐患排查治理常态化工作机制，持续巩固安全生产稳定局面。加强社区建设，提升管理服务水平。坚持依法治市，完成“六五”普法各项任务。加强社会治安综合治理，建立“一村一警”长效机制，有效防范和化解各类矛盾和问题，确保社会和谐稳定。

加强新形势下国防教育和国防后备力量建设，支持驻平解放军、武警部队、预备役部队、民兵和人民防空建设，争创全国双拥模范城“五连冠”。支持工会、共青团、妇联等人民团体发挥桥梁纽带作用，注重发挥社团作用。加强统计工作，搞好全国1%人口抽样调查。做好对台、民族、宗教、史志、档案、地震等工作。

科学编制“十三五”规划。广泛吸收人大代表、政协委员等各界意见，用改革的精神、创新的理念和科学的方法，谋划好重大基础设施项目、重大经济发展项目和重大民生工程项目，争取一批重大项目列入国家和省规划。

各位代表！

做好新常态下政府工作，必须持续强化责任意识，提升履职能力，弘扬务实作风，以建设法治政府和服务型政府为重点，切实加强自身建设。新的一年，各级政府要牢固树立法治理念，健全依法决策机制，建立法律顾问制度，提升依法履职能力。加快转变政府职能，整体推进服务型行政执法。严格执行人大及其常委会的决议决定，自觉接受法律监督和工作监督，主动接受政协民主监督，认真办理人大代表建议和政协提案，虚心接受舆论监督和社会监督，切实推进政府信息公开，让权力在阳光下运行。坚定不移推进反腐倡廉建设，厉行勤俭节约，反对铺张浪费，做到有腐必惩、有贪必肃。巩固扩大群众路线教育实践活动成果，深入开展专题调研、现场办公，强化督察、严格考核，推动各项工作落到实处。努力通过一件件小事的办理、一件件实事的落实、一件件难事的解决，赢得人民群众的更多信任，获得人民群众的更大支持！

各位代表！

职责所系，不容丝毫懈怠；重任在肩，更需策马扬鞭。让我们在市委的坚强领导下，紧紧依靠全市人民，以更大的责任担当、更实的工作作风，凝神聚力、团结奋进，为加快建设资源型城市可持续发展示范区、全面建成小康社会而努力奋斗！
